# A phylogenetic analysis of Bovine Viral Diarrhoea Virus (BVDV) isolates from six different regions of the UK and links to animal movement data

**DOI:** 10.1186/1297-9716-44-43

**Published:** 2013-06-19

**Authors:** Richard E Booth, Carole J Thomas, Laila MR El-Attar, George Gunn, Joe Brownlie

**Affiliations:** 1 Department of Pathology and Infectious Disease, Royal Veterinary College, Hawkshead Lane, North Mymms, Hertfordshire AL9 7TA, United Kingdom; 2 Epidemiology Research Unit, SAC, Drummondhill, Stratherrick Road, Inverness IV2 4JZ, United Kingdom

## Abstract

Bovine Viral Diarrhoea Virus (BVDV) is a pestivirus which infects cattle populations worldwide and is recognised as a significant source of economic loss through its impact on health and productivity. Studies investigating the molecular epidemiology of BVDV can give invaluable information about the diversity of viral strains present in a population and this, in turn, can inform control programs, drive vaccine development and determine likely infection sources. The current study investigated 104 viral isolates from forty farms across the UK. Through phylogenetic and nucleotide sequence analysis of the 5′UTR and N^pro^ regions of the isolates investigated, it was determined that BVDV 1a was the predominant sub-genotype. However, BVDV 1b, 1e and 1i were also identified and, for the first time in the UK, BVDV 1d. Through analysis of animal movement data alongside the phylogenetic analysis of these BVD isolates, it was possible to link animal movements to the viral isolates present on several premises and, for the first time, begin to elucidate the routes of viral transmission. With further work, this type of analysis would enable accurate determination and quantification of the true biosecurity risk factors associated with BVDV transmission.

## Introduction

Bovine Viral Diarrhoea Virus (BVDV) infects cattle worldwide and is a cause of significant losses in both beef and dairy systems. The virus is a member of the pestivirus genus in the *Flaviviridae* family. It is a positive sense, single stranded RNA virus of approximately 12.5 kb in length [1]. Pestivirus genomes are flanked by 5′ and 3′ untranslated regions (5′UTR, 3′UTR) and encode 11–12 structural and non-structural proteins (N^pro^, C, E^rns^, E1, E2, P7, NS2/3, NS4A, NS4B, NS5A, NS4B). The genus is currently comprised of four recognised species; BVDV-1, BVDV-2, Border Disease Virus (BDV) and Classical Swine Fever Virus (CSFV) and four proposed “atypical” species Giraffe, “HoBi”, Pronghorn Antelope and Bungowannah [2-7].

Pestiviruses are highly variable both antigenically and genetically, hence each species is further classified. BVDV is currently divided into two genotypes [8]. More recent molecular analysis, primarily based upon sequence analysis of the 5′UTR and the non-structural N^pro^ gene, has resulted in further sub-classification such that BVDV genotype 1 currently contains twelve sub-genotypes; BVDV 1a-k plus Deer [9,10]. Six further sub-genotypes of BVDV1 have been proposed (1l, 1m, 1n, 1o and 1p with BVDV 1l being designated twice for diverse sets of isolates) although the literature concerning some of these members requires further clarification and is discussed in detail later [3,11-15]. BVDV 2 is currently separated into at least two sub-genotypes [16-18].

Studies investigating the molecular epidemiology of BVDV can provide invaluable information about the diversity of viral strains present in a population and, in turn, inform control programs, drive vaccine development and determine likely infection sources. In a survey of bovine pestiviruses published in 1999, 62 BVD viral isolates across England and Wales were sequenced and were predominantly found to be BVDV 1a although BVDV 1b and 1i were also identified [10,19]. A more recent study conducted between 2004 and 2009 indicated that whilst BVDV 1a was still the predominant sub-genotype, and that 1b and 1i were still circulating, the genetic diversity of BVDV in England and Wales has increased further to include BVDV 1e and 1f [20]. Here, we provide results of a further phylogenetic analysis of pestiviruses isolated from cattle across several regions of the UK including some of the most cattle dense regions in England with findings that largely support those of Strong et al. [20]. Furthermore, we have analysed cattle movement information for a number of farms participating in this study. For the first time with BVDV, we have demonstrated that it is possible to elucidate potential sources and routes of infection if animal movements are correlated with phylogenetic analyses.

## Materials and methods

### Farm distribution for sample collection

**Figure 1:**
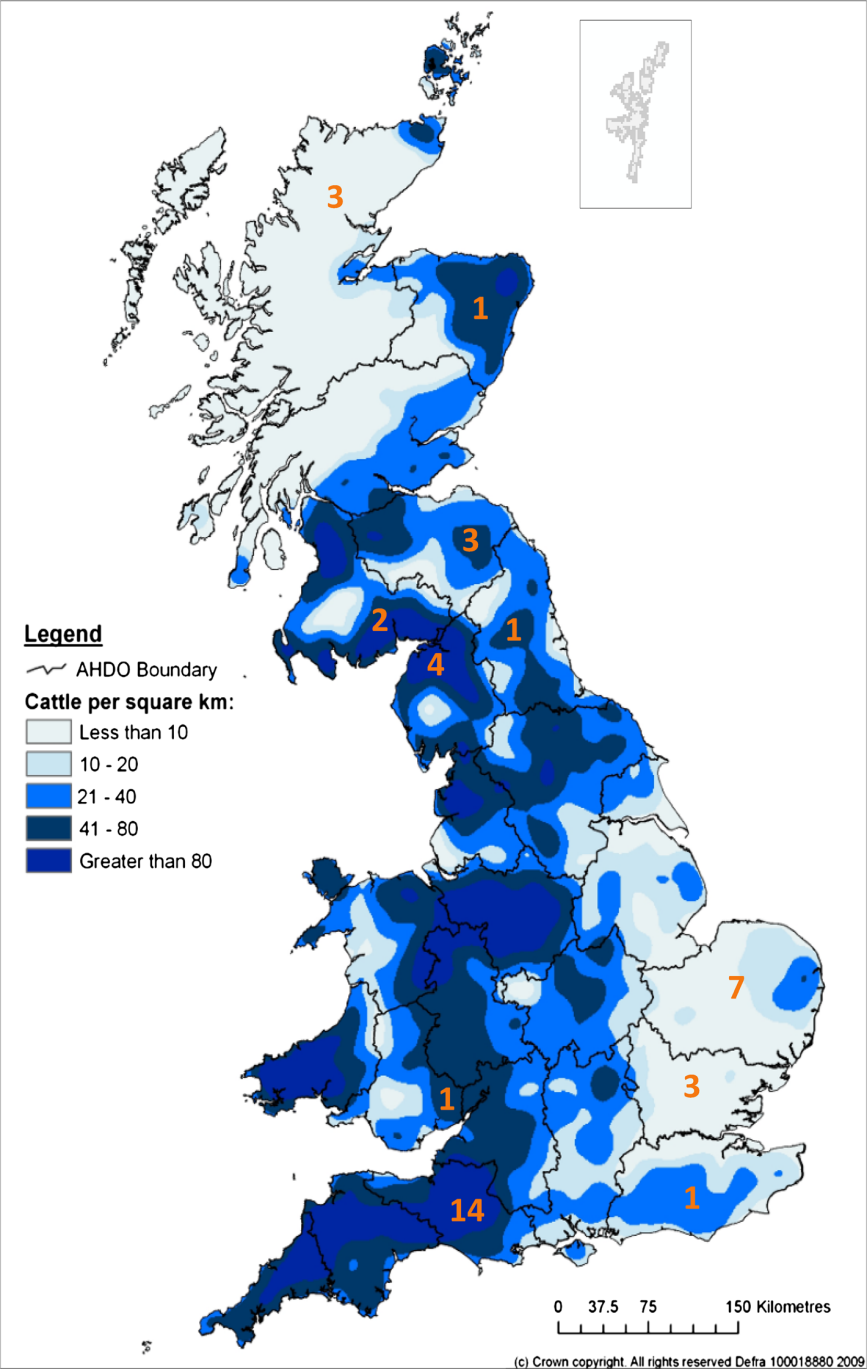
**Regional distribution of infected farms investigated per Animal Health Divisional Office region relative to the cattle density in Great Britain in 2008.** The cattle population density and Animal Health Divisional Office (AHDO) Boundaries illustrated in Great Britain are as of 1^st^ June 2008 (“The Cattle Book 2008” © Crown copyright. Reproduced with kind permission of Ordnance Survey).

Samples were collected from PI animals identified on farms that were screened across six regions of the UK between 2006 and 2011 (see Table 1 and Figure 1). In summary, four farms were sampled in South-east England, fourteen in South-west England, seven in East Anglia, five in Northern England, one in Wales and nine in Eastern Scotland. Samples were labelled with “Farm number – PI number” such that the farm of origin and order of PI identification is consistent throughout. For example, Isolate 33–3 is Farm 33-third PI identified. This numbering also allows cross referencing to the farms previously described by Booth and Brownlie [21].

**Table 1:** Regional distribution and details of BVDV infected premises investigated in this study

Farm number*	**Region (by local AHDO)**	**Dairy/Beef**	**Number of PI samples received for sequencing**	**BVDV scheme**
**1**	Taunton	Dairy	3	RVC
**2**	Taunton	Dairy	9	RVC
**5**	Taunton	Dairy	1	RVC
**15**	Taunton	Dairy	1	RVC
**18**	Taunton	Dairy	6	RVC
**19**	Taunton	Dairy	1	RVC
**20**	Taunton	Dairy	3	RVC
**26**	Taunton	Dairy	3	RVC
**27**	Taunton	Dairy	1	RVC
**33**	Taunton	Dairy	2	RVC
**34**	Taunton	Dairy	1	RVC
**37**	Taunton	Dairy	5	RVC
**38**	Taunton	Dairy	1	RVC
**40**	Chelmsford	Dairy	8	RVC
**41**	Chelmsford	Beef	4	RVC
**42**	Reigate	Dairy	2	RVC
**43**	Newcastle	Dairy	8	SAC, inverness
**44**	Galashiels	Dairy	2	SAC, inverness
**45**	Carlisle	Dairy	1	SAC, inverness
**46**	Carlisle	Dairy	1	SAC, inverness
**47**	Carlisle	Dairy	1	SAC, inverness
**48**	Inverness	Dairy	10	SAC, inverness
**49**	Galashiels	Dairy	1	SAC, inverness
**50**	Galashiels	Dairy	2	SAC, inverness
**56**	Inverurie	Dairy	5	Lab submission
**57**	Bury St Edmunds	Beef	2	EA
**58**	Bury St Edmunds	Dairy	2	EA
**59**	Bury St Edmunds	Dairy	2	EA
**60**	Bury St Edmunds	Beef	1	EA
**61**	Bury St Edmunds	Dairy	1	EA
**62**	Bury St Edmunds	Dairy	3	EA
**63**	Ayr	Unknown	1	Lab submission
**64**	Ayr	Unknown	1	Lab submission
**65**	Inverurie	Unknown	1	Lab submission
**66**	Inverurie	Unknown	1	Lab submission
**67**	Carmarthen	Dairy	2	Practice submission
**68**	Taunton	Dairy	1	Practice submission
**69**	Carlisle	Unknown	1	Practice submission
**70**	Bury St Edmunds	Dairy	2	Practice submission
**71**	Chelmsford	Dairy	1	Practice submission

*Farms 3, 4, 6–14, 16, 17, 21–25, 28–32, 35, 36, 39 were uninfected farms that were part of the Somerset pilot scheme described by Booth and Brownlie [21] hence do not appear in this table of infected premises. Farms 51–55 were infected members of a Scottish pilot scheme (SAC, Inverness) and samples have not been processed yet. Animal Health Divisional Offices (AHDO) are those in existence in 2008 and correspond with the boundaries marked in Figure 1.

### Virus RNA isolation and cDNA synthesis

Blood samples collected from PI animals were either heparinised or clotted (this was at the discretion of the clinician taking the sample). RNA was isolated from heparinised whole blood using a QIAamp RNA Blood Mini Kit (QIAGEN Ltd., Manchester, UK) and from serum using a QIAGEN QIAamp Viral RNA Kit (QIAGEN Ltd.) according to the manufacturer’s instructions.

cDNA was synthesised from isolated RNA using Invitrogen Superscript II reverse transcription kit (Invitrogen, Paisley, UK). For each cDNA reaction, 5 μL of RNA was mixed with 3 μL of pd(N)_6_ Random Hexamers (GE Healthcare, Hatfield, UK) and incubated at 70°C for 10 min. 8 μL of 5× Superscript II First Strand Buffer (Invitrogen), 4 μL of 0.1M DTT (Invitrogen), 2 μL of 10 mM dNTPs (Promega, Southampton, UK), 16 μL nuclease-free dH_2_O and 1 μL of 40 U/μL RNasin (Promega) was then added. After incubating the mixture at 42°C for 2 min, 1 μL of 200 U/μL Superscript II Reverse Transcriptase enzyme (Invitrogen) was then added to each sample and the incubation continued for a further 50 min followed by 10 min at 72°C. RNA samples were stored at −70°C and cDNA samples were stored at −20°C for future use.

### RT-PCR and DNA sequencing

For each viral isolate, a 288bp and 428bp region of the 5′UTR and N^pro^ regions respectively were amplified. At least two PCR reactions for each region, using a high fidelity *Pfu* DNA polymerase enzyme (Stratagene, Stockport, UK), were performed on each sample. If consensus sequence could not be obtained from sequence analysis of two PCR reactions, further reactions and sequencing were performed in order to achieve this. Primers 324 and 326 were used for amplification of a 288bp region of the 5′ UTR as described by Vilcek et al. [22]. Primers BD1 [23] and a modified version of BD3 [10], named BD3A, were used for amplification of a 428bp region of N^pro^ under the same conditions described by Vilcek et al. [10] for the BD1/BD3 pair. BD3A was designed in house and the sequence of the modified primer is: 5′- CAT CCA TCT ATR CAY AYA TAA ATR TGG TAC - 3′.

For PCR reactions, the following was setup; 2 μL template cDNA, 5 μL Forward primer (10 pmol/μL), 5 μL Reverse primer (10 pmol/μL), 5 μL 10 × *Pfu* DNA polymerase reaction buffer (Stratagene), 1 μL 10mM dNTP, 0.5 μL *Pfu* DNA polymerase 2.5 U/μL (Stratagene) and 31.5 μL dH_2_0. The PCR conditions were as follows: 94°C for 3 min initial denaturing followed by 30 cycles of 94°C for 1 min, 52°C for 1 min, 72°C for 1 min and then final elongation at 72°C for 10 min. Amplicons of the expected sizes (288bp for 5′UTR and 428bp for N^pro^) were gel purified or PCR purified (based on whether or not a single individual amplicon was observed) using QIAquick /PCRGel Extraction Kit (QIAGEN). Purified PCR products were sequenced by QIAGEN Genomic Services (Hilden, Germany) using Dye Terminator cycle sequencing performed with the ABI PRISM BiG DYE V3.1 Terminator Cycle Sequencing. At least two independent PCR reactions were sequenced for each sample using forward and reverse primers 324/326 or BD1/BD3A for 5′UTR and N^pro^ respectively.

### DNA sequencing and phylogenetic analysis

The sequences received were initially analysed in Vector NTI Version 11 (Invitrogen) and consensus sequences were assembled in the Contig Express feature of Vector NTI where they were checked for discrepancies. Primer sequences were clipped from each consensus prior to phylogenetic analysis conducted using *MEGA* version 5 [24]. 5′UTR and N^pro^ sequences in FASTA format were imported into MEGA 5 and sequence alignments performed using ClustalW. Phylogenetic analysis was performed using the Neighbour Joining Method [25] and evolutionary distances were calculated using the Kimura 2-parameter method [26]. Bootstrap analysis of the resultant tree was performed using 1000 replicates with deletion of gaps in the alignment data. Bootstrap figures >70% are reported [27].

### Cattle movement analysis

Sixty-two farms gave permission to obtain animal movement records relating to their premises. This network included thirty-one farms from which PI samples were genotyped, twenty-six farms where BVDV surveillance was conducted but no PI animals identified and five farms on which PI animals were identified but genotyping not yet performed. These are listed as Farms 1–62 in Table 1. Animal movements recorded by the British Cattle Movement Service (BCMS) were sourced from the Department for Environment, Food and Rural Affairs (DEFRA) veterinary surveillance group “Rapid Analysis and Detection of Animal-related Risks” (RADAR). RADAR supplied movement data for Farms 1–62 to include cattle movements between study farms, through intermediate premises which were not in the study (anonymous), markets and showgrounds. Movement data was supplied in text file format which was imported into Microsoft Access. Movement details were extracted from the database through a series of queries producing matrices of movements between relevant premises. Movement links were scrutinised alongside the phylogenetic analysis to assess links between virus strain and animal movements.

## Results

### Distribution of regions sampled

One hundred and four viral isolates were collected from PI cattle on forty farms across the UK. The geographical location of the farms and the way in which the samples were submitted is summarised in Table 1. Samples from ten farms were submitted to the BVDV research group at The Royal Veterinary College (RVC) by individual practitioners or laboratories. The remaining samples came from members of organised BVDV control programmes. Six farms surveyed across East Anglia were members of an eradication programme promoted by Holstein UK (HUK) which, received part funding from the English Beef and Lamb Executive (EBLEX). Twenty-four of the farms surveyed were involved in an eradication scheme set up jointly by RVC and the Scottish Agricultural College (SAC) which recruited farms in Somerset, Southeast England, Northern England and Eastern Scotland. The Somerset/South-Eastern eradication programme is described in detail by Booth and Brownlie [21]. The distribution of farms sampled compared to UK cattle density is illustrated in Figure 1 and whilst some cattle dense regions were sampled, it should be noted that the sampling cannot be considered representative of Wales, the Welsh borders or the far southwest of England.

### Phylogenetic and sequence analysis

Of the 104 viral isolates investigated, 101/104 5′UTR and 98/104 N^pro^ sequences were obtained for phylogenetic analysis and sequence comparison. Table 2 gives details of the number of PIs investigated on each infected farm and the sub-genotypes identified. The majority of viral isolates, 85% (88/104), were typed as BVDV 1a based upon analysis of both 5′UTR and N^pro^ and comparison to a set of reference strains detailed in Table 3. However, also identified were BVDV types 1b (Farms 18 and 71), 1d (Farm 67), 1e (Farms 1 and 68) and 1i (Farms 58 and 69). There was no noticeable geographical distribution of isolates with BVDV 1a being identified in all regions sampled except Wales where only one farm had been sampled.

**Table 2:** Strain allocation table

**Sample**	**Closest 5′UTR reference strain**	**% Identity to 5′UTR reference strain**	**Closest N**^**pro **^**reference strain**	**% Identity to N**^**pro **^**reference strain**
**1-1**	1e	95	1a	83
**1-2**	1e	95	1a	83
**1-3**	1e	95	-	-
**2-1**	1a	98	1a	92
**2-5**	1a	98	1a	92
**2-6**	1a	98	1a	92
**2-7**	1a	98	1a	92
**2-8**	1a	96	1a	90
**2-9**	1a	98	1a	92
**2-10**	1a	96	1a	90
**2-11**	1a	97	1a	92
**2-12**	1a	96	1a	90
**5-1**	1a	95	1a	91
15-1*	1a/1j	95 to both	1a	90
**18-1**	1b	97	1b	95
**18-2**	1b	97	1b	95
**18-3**	1b	97	1b	95
**18-4**	1b	97	1b	95
**18-5**	1b	97	1b	95
**18-6**	1b	97	1b	95
**19-1**	-	-	1a	93
20-1*	1a/1j	96 to both	1a	92
20-2*	1a/1j	96 to both	1a	91
20-3*	1a/1j	96 to both	1a	91
**26-1**^**+**^	1a	96	1a	91
**26-2**^**+**^	1a	96	1a	91
**26-3**^**+**^	1a	96	1a	91
27-1*	1a/1j	95 to both	1a	92
**33-1**^**#**^	1a	94	1a	91
**33-3**^**#**^	1a	95	1a	92
**34-1**^**#**^	1a	95	1a	92
37-1*^#^	1a/1j	95 to both	1a	88
37-2*^#^	1a/1j	95 to both	1a	88
37-3*^#^	1a/1j	95 to both	1a	88
37-4*^#^	1a/1j	95 to both	1a	88
37-5*^#^	1a/1j	95 to both	1a	88
**38-1**	1a	97	1a	92
**40-1**	1a	99	1a	92
**40-2**	1a	96	1a	92
**40-3**	1a	99	1a	92
**40-4**	1a	96	1a	92
**40-5**	1a	96	1a	93
**40-6**	1a	99	1a	92
**40-7**	1a	96	1a	93
**40-8**	1a	96	1a	92
**41-1**	1a	95	1a	92
**41-2**	1a	95	1a	92
**41-3**	1a	95	1a	92
**41-4**	1a	95	1a	92
**42-1^**	1j	95	1a	81
**42-2^**	1j	95	-	-
**43-1**	-	-	1a	92
**43-2**^**+**^	1a	96	1a	92
**43-3**^**+**^	1a	96	1a	92
**43-4**^**+**^	1a	96	1a	92
**43-5**^**+**^	1a	96	1a	92
**43-6**^**+**^	1a	96	1a	92
**43-7**^**+**^	1a	96	1a	92
**43-8**^**+**^	1a	96	1a	92
**44-1**^**+#**^	1a	95	1a	93
44-2*^#^	1a/1j	94 to both	1a	93
**45-1**^**#**^	1a	95	1a	92
**46-1**	1a	96	1a	90
**47-1**	1a	97	1a	92
**48-1**	1a	96	1a	91
**48-2**	1a	96	1a	91
**48-3**	1a	96	1a	91
**48-4**	1a	96	1a	91
**48-5**	1a	96	-	-
**48-6**	1a	96	1a	91
**48-7**	1a	96	1a	91
**48-8**	1a	96	1a	91
**48-9**	1a	96	1a	91
**48-10**	1a	96	1a	91
**49-3**	1a	96	1a	93
**50-1**	1a	98	1a	92
**50-2**	1a	98	1a	91
56-1*^#^	1a/1j	94 to both	1a	92
56-2*^#^	1a/1j	94 to both	1a	92
**56-3**^**+#**^	1a	94	1a	92
56-4*^#^	1a/1j	94 to both	1a	92
56-5*^#^	1a/1j	94 to both	1a	92
**57-1**	1a	97	1a	92
**57-2**	1a	97	1a	92
**58-1**	1i	98	1i	98
**58-2**	1i	98	1i	98
**59-1**^**+**^	1a	96	1a	92
**59-2**	1a	98	1a	92
60-1*	1a/1j	94 to both	1a	90
**61-1**^**+**^	1a	96	1a	92
**62-1**	-	-	1a	92
62-2*^#^	1a/1j	94 to both	-	-
62-3*^#^	1a/1j	94 to both	1a	91
63-1*	1c/1a/1j	95 to all	1a	93
**64-1**^**+**^	1a	96	1a	84
**65-1**^**+**^	1a	95	1a	92
**66-1**	1a	97	1a	93
**67-1**	1d	97	1d	90
**67-2**	1d	97	1d	90
**68-1**	1e	98	-	-
**69-1**	1i	97	-	-
**70-1**	1a	95	1a	92
**70-2**	1a	95	1a	92
**71-1**	1b	98	1b	94

The table indicates the percentage identity of the closest reference strain to the field isolate investigated. Reference strains used are described in Table 3. Where discrepancies or high nucleotide identity occurs with multiple reference strains, this is noted as follows: * indicates isolates with equal 5′UTR nucleotide sequence identity to both BVDV 1a and 1j reference strains, ^+^ indicates isolates with 1% less 5′UTR nucleotide identity to BVDV 1j reference strains than to 1a reference strains, ^ indicates isolates with 1% higher 5′UTR identity to BVDV 1j reference strains than to either 1a or 1c reference strains, ^#^ indicates isolates with 1% less 5′UTR nucleotide identity to BVDV 1c reference strains than to 1a reference strains, – indicates that no sequence was obtained. (GenBank Accession numbers for the 5′UTR and N^pro^ regions sequenced for this paper are KF023272-KF023470).

**Table 3:** Reference strains used in this publication

**Reference strain name**	**Type**	**N**^**pro **^**& 5′UTR available?**	**5′UTR GenBank accession number**	**N**^**pro **^**GenBank accession number**	**Reference**
**NADL**	1a	Yes	M31182^+^	M31182^+^	[28]
**SD1**	1a	Yes	M96751^+^	M96751^+^	[29]
**28-1**	1a	No^#^	AF298061	-	[10]
**Osloss**	1b	Yes	M96687^+^	M96687^+^	[30]
**24-15**	1b	Yes	AF298060	AF287280	[10]
**P**	1b	Yes	AF298070	AF287288	[10]
**T**	1b	yes	AF298072	AF287289	[10]
**Bega**	1c	Yes	AF049221^+^	AF049221^+^	Direct submission to GenBank
**B666 Mogilla**	1c	No	JQ743605	-	[31]
**B701 Crookwell**	1c	No	JQ743606	-	[31]
**B702 Grafton**	1c	No	JQ743607	-	[31]
**16-111**	1d	No	AF298056	-	[10]
**F**	1d	Yes	AF298065	AF287284	[10]
**871**	1d	No	-	AF144462	[16]
**721**	1d	No	-	AF144463	[16]
**10-84**	1e	No^#^	AF298054	-	[10]
**20-V661-2**	1e	No^#^	AF298058	-	[10]
**3186V6**	1e	Yes	AF298062	AF287282	[10]
**26-V639**	1e	No	-	AF287281	[10]
**J**	1f	Yes	AF298067	AF287286	[10]
**R**	1f	No	AF298071	-	[10]
**W**	1f	Yes	AF298073	AF287290	[10]
**A**	1g	Yes	AF298064	AF287283	[10]
**L**	1g	Yes	AF298069	AF287287	[10]
**G**	1h	Yes	AF298066	AF287285	[10]
**KM**	1h	No	AF298068	-	[10]
**23-15**	1i	Yes	AF298059	AF287279	[10]
**KS861-ncp**	1J	Yes	AB078950^+^	AB078950^+^	[32]
**Deer_GB1**	1J	No	-	U80902	[5]
**M1515A**	1J*	No	U97429	-	[33]
**M065B**	1J*	No	U97409	-	[33]
**SuwaCp**	1k*	No	AF117699	-	[34]
**Rebe**	1k*	No	AF299317	-	Direct submission to GenBank
**71-16**	1l^	Yes	Supplied by Prof. Vilcek	Supplied by Prof. Vilcek	[11]
**71-03**	1l^	Yes	Supplied by Prof. Vilcek	Supplied by Prof. Vilcek	[11]
**71-15**	1l^	Yes	Supplied by Prof. Vilcek	Supplied by Prof. Vilcek	[11]
**ZM-95**	1m	Yes	AF526381^+^	AF526381^+^	[13]
**890**	2	Yes	U18059^+^	U18059^+^	[35]
**15-103**	2	No	AF298055		[10]
**4-5174**	2	No	AF298063		[10]

Reference strains used, their GenBank accession numbers and whether both 5′UTR and N^pro^ regions are available are indicated. Where relevant, the publications originally detailing the strains are noted. ^#^N^pro^ typed in Vilcek et al. [10], but no sequence available in GenBank. *subtype classified by Vilcek et al. [9]. ^Sequences classified by Jackova et al. [11] and sequence information obtained directly from Professor Vilcek. ^+^Whole genome sequence extracted from GenBank.

**Figure 2:**
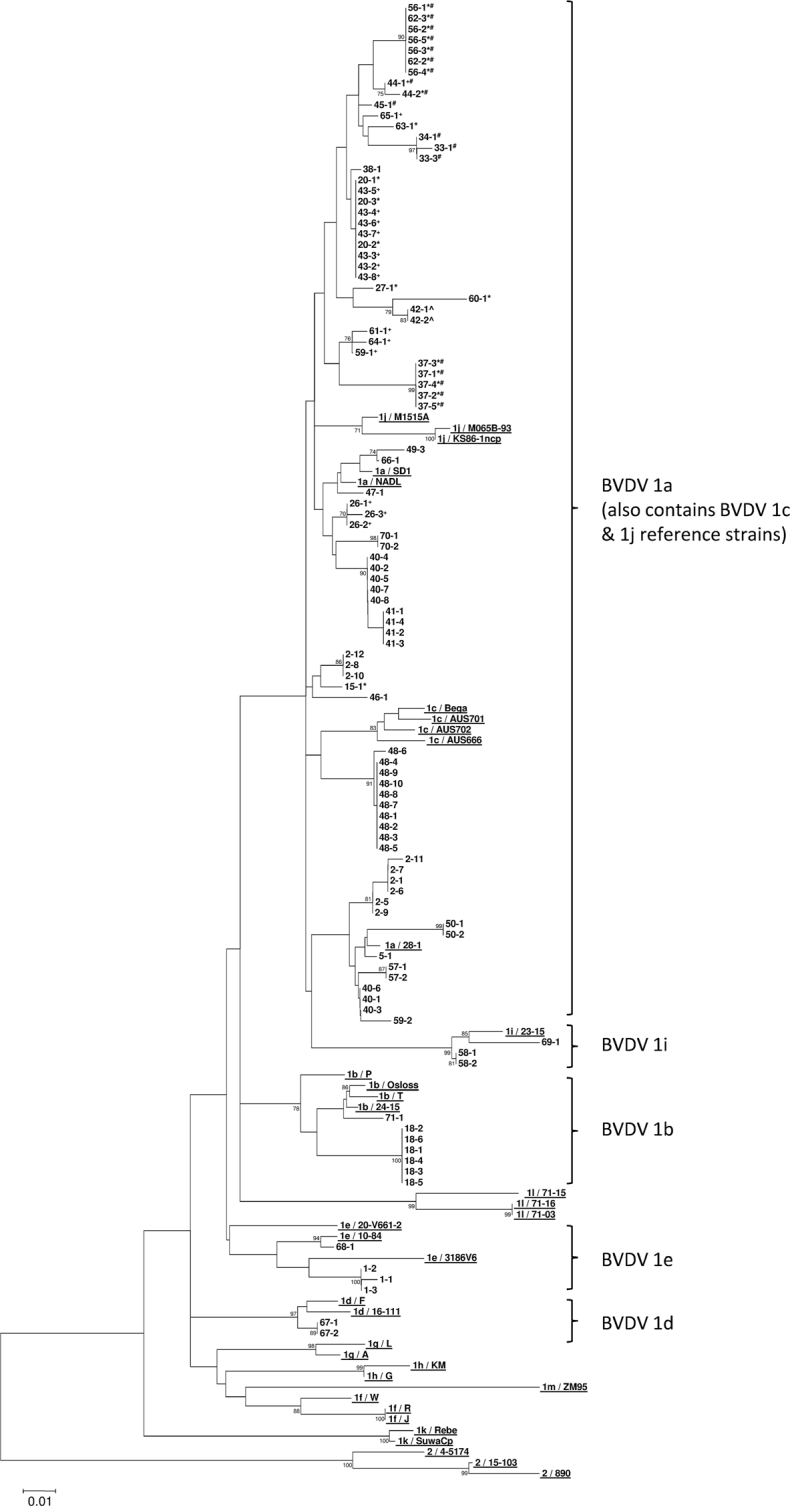
**Phylogenetic tree of a 245bp region of the 5′UTR.** All field isolates are denoted by “Farm Number – PI Number”. * indicates isolates with equal 5′UTR nucleotide sequence identity to both BVDV 1a and 1j reference strains, ^+^ indicates isolates with 1% less 5′UTR nucleotide identity to BVDV 1j reference strains than to 1a reference strains, ^ indicates isolates with 1% higher 5′UTR identity to BVDV 1j reference strains than to either 1a or 1c reference strains, ^#^ indicates isolates with 1% less 5′UTR nucleotide identity to BVDV 1c reference strains than to 1a reference strains. Thirty six reference strains are included (underlined) each beginning with the genotype and sub-genotype to which they belong (1a-1m and 2) followed by their name; further details can be found in Table 3. Bootstrap figures supported by >70% of 1000 replicates are indicated.

**Figure 3:**
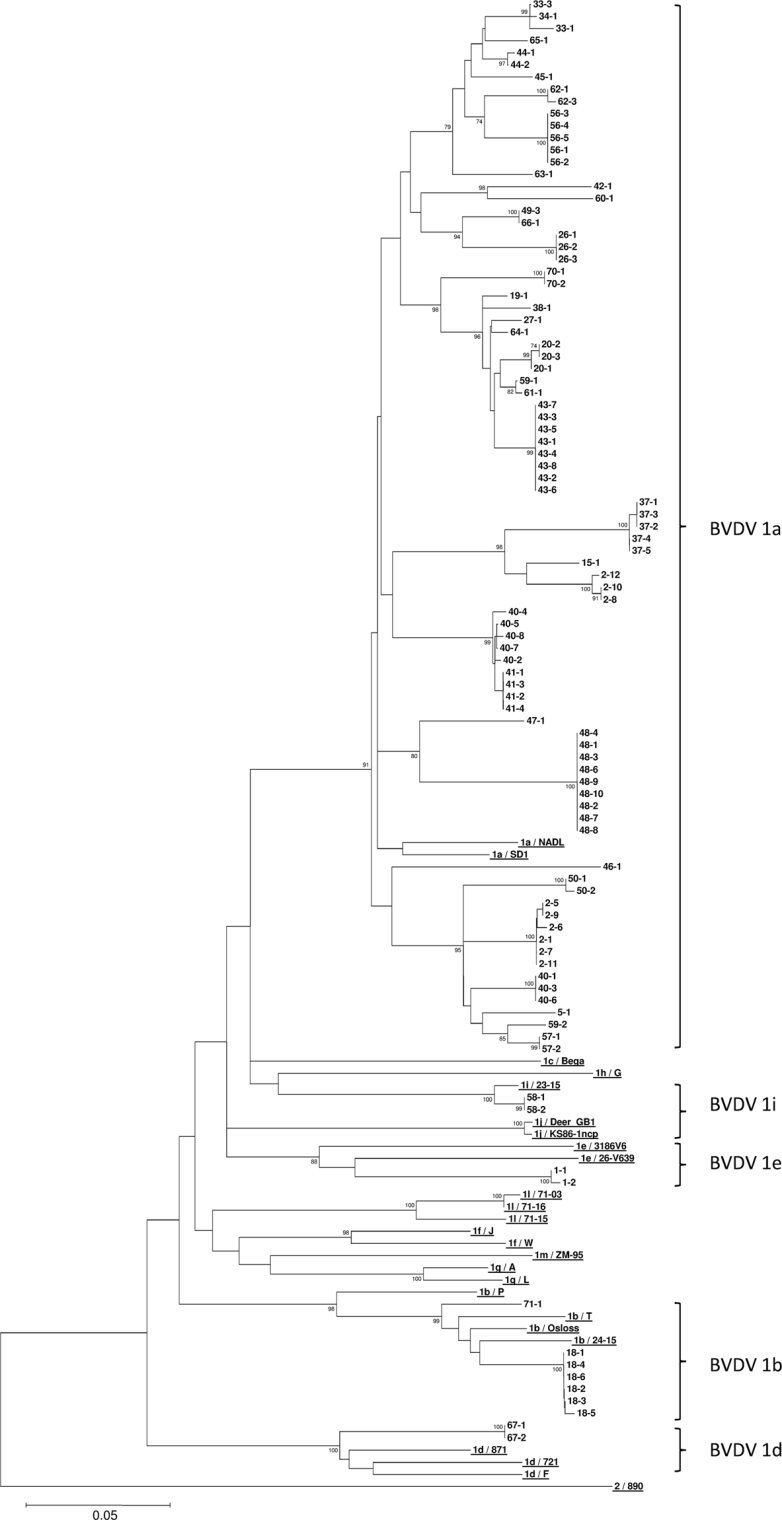
**Phylogenetic tree of a 380bp region of N**^**pro**^**.** All field isolates are denoted by “Farm Number – PI Number”. Twenty five reference strains are included (underlined) each beginning with the genotype and sub-genotype to which they belong (1a-1m and 2) followed by their name; further details can be found in Table 3. Bootstrap figures supported by >70% of 1000 replicates are indicated.

For the viral isolates investigated, the apparent sub-genotypes indicated by the 5′UTR and N^pro^ phylogenetic trees in Figures 2 and 3 respectively were largely supported by nucleotide sequence comparisons to the reference strains in Table 3. However, several issues that confused isolate sub-genotype classification became apparent when using the 5′UTR alone and this is noted in Table 2. The analysis of N^pro^, shown in Table 2, provided a much clearer definition of the sub-genotypes investigated. The only instance of confusion generated through N^pro^ analysis occurred for isolates 1–1 and 1–2, which both had the highest identity (95%) to the BVDV 1e 5′UTR reference strains, yet upon N^pro^ analysis were most identical (83%) to the BVDV 1a reference strain SD1. However, removal of SD1 from the N^pro^ analysis left isolates 1–1 and 1–2 with closest identity (82%) to BVDV 1e, 1i, 1j and 1m reference strains and only 81% identity to NADL (1a); hence, isolates from Farm 1 clustered phylogenetically with BVDV 1e in both Figures 2 and 3 (5′UTR and N^pro^ respectively).

### Movement data analysis

**Figure 4:**
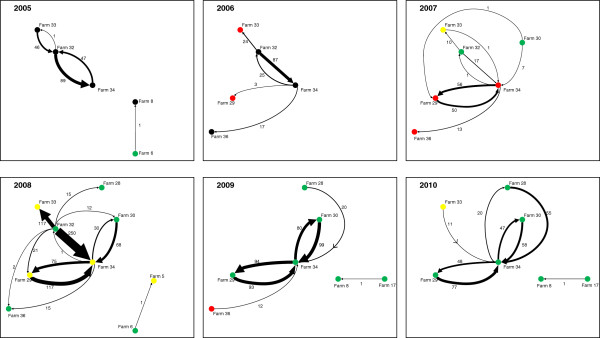
**Farm status and animal movement matrix between study Farms 1–42 (2005–2010):** Each coloured node represents an individual farm. Farm number is stated alongside the node. The status of each farm is defined by the colour of its node such that: a “Black Node” represents a farm of unknown status, a “Green Node” represents a BVDV free farm, a “Red Node” represents a BVDV infected farm and a “Yellow Node” represents a farm that is currently eradicating BVDV. Numbers of animal movements are indicated alongside the arrow that illustrating the direction of movement. The arrow line width is proportional to the number of movements. Geographic position and relationships are not represented in the figure.

Movement data was available for Farms 1–62 in Table 1 for the period January 2005 to December 2010. Farms 63–71 had not given permission for analysis of their animal movement data. In addition to cattle movement data, the yearly infection status was available for Farms 1–42 from the study conducted by Booth and Brownlie [21], thus enabling the construction of an animal movement network linked to farm BVD status between 2005 and 2010 (Figure 4). Indirect links between premises involved, such as animal movements through other farms, markets or showgrounds were infrequent with the latter being identified as the only indirect animal movement link between farms in this study (see below). From analysis of the sequence data and animal movements, it was possible to describe three situations regarding the epidemiology of BVDV transmission.

1. Same farm, same isolate: This situation is demonstrated in both Figures 2 and 3 for all farms in this study where more than one PI isolate was investigated, except for Farms 2, 40 and 59 (see point 2 below). Typically, isolates from PIs born on one premise were seen to cluster phylogenetically with others from the same farm indicating that the infecting strains have very high identity. Analysis of the nucleotide identity matrices for the 5′UTR and N^pro^ sequences where more than one PI was identified on the same farm revealed that they shared 99%–100% nucleotide identity in both regions investigated.

2. Same farm, different isolate: In some instances it is evident that two isolates have caused infection on the same farm; Farms 2, 40 and 59 are examples of this. For Farms 2 and 40, two clusters of PIs are present for each farm in the phylogenetic trees in Figures 2 and 3. Nucleotide sequence analysis for these isolates reveals that for both farms, isolates share 99%–100% identity in both the 5′UTR and N^pro^ regions within each PI cluster. However, between clusters on the same farm, identity in the 5′UTR and N^pro^ regions ranges from 95%–96% and 88%–90% respectively. For Farm 59, only two PIs were identified and these shared nucleotide sequence identities in the 5′UTR and N^pro^ regions of only 95% and 91% respectively thus grouped separately from each other upon phylogenetic analysis (Figures 2 and 3).

3. Different farms, same isolate: This instance appears to occur on several farms if the 5′UTR in Figure 2 is considered alone however, the results indicate that N^pro^ also needs to be considered to make conclusions of this nature. Viral isolates from PIs on Farms 40 and 41 (isolates share 99%–100% 5′UTR nucleotide identity), Farms 20 and 43 (isolates share 99% 5′UTR nucleotide identity), Farms 56 and 62 (isolates share 99%–100% 5′UTR nucleotide identity) and Farms 33 & 34 (isolates share 100% 5′UTR nucleotide identity) are seen to cluster as distinct groups in Figure 2. The N^pro^ phylogenetic tree in Figure 3, confirms the links between Farms 33 & 34 and 40 & 41 with isolates clustering together and achieving 99%–100% nucleotide identity in this region. The links are less well supported by N^pro^ phylogenetic analysis of Farms 20 & 43 and 56 & 62 with isolates from these farm groups returning 97% and 96% N^pro^ nucleotide identity respectively. Whilst these identities are still high, the clustering of isolates from these farms is less convincing in Figure 3 when compared to that seen in Figure 2. Geographically, none of these farms share borders (data not shown); however, the possible animal/material links between farms that presented potential biosecurity pathways for virus transmission are highlighted below.

Farms 33 and 34 have been linked by direct animal movements (1 animal in 2007 and 11 animals in 2010) and also through indirect animal movements through Farm 32 throughout the study period. Figure 4 indicates that BVDV infection was detected on Farms 33 and 34 in 2006 and 2007 respectively thus the animal movements in 2010 are unlikely to be responsible for transmission of infection between these farms. It is feasible that the one movement noted in 2007 could have been responsible for the presence of the same BVDV isolate on both premises. However, there are five further premises involved in the movement network with Farms 33 and 34 in Figure 4 (Farms 28, 29, 30, 32 and 36). It can be noted that multiple movements occur between Farm 32 and 33 and Farm 32 and 34. It is quite possible that infected animals have passed between Farms 34 and 33 through Farm 32. However, it is curious that infection was not detected on Farm 32 at any point. Further to this, it can also be noted that BVDV infection was detected on Farms 29 and 36 in 2007 with the former testing bulk milk PCR positive and the latter testing positive in youngstock for BVD antibody in 2007 (data not shown). PI searches were delayed on these farms due to management changes and thus it is believed that any PIs present left these premises prior to whole herd investigations, thus viral isolates were not available from these farms sub-genotyping. The involvement of infected farms in this network illustrates the risk to the other farms involved; biosecurity advice was given to all farms involved in order to mitigate this. Direct animal movements were also noted from Farm 50 to 49 throughout 2006–2010 and from Farm 49 to 50 in 2007 (data not shown), yet Figures 2 and 3 do not indicate a viral isolate that is common to both farms suggesting that they had different infection sources and that one had not infected the other.

Whilst the isolates from Farms 40 and 41 appear linked when analysing both the 5′UTR and N^pro^ phylogenetic trees, they were not linked by direct animal movements between their premises. Despite this, the high identity noted in both the 5′UTR and N^pro^ regions of viral isolates from these farms supports the theory that there is a common isolate in both units. It is known to the authors that the owners of Farms 40 and 41 exhibit animals at the same agricultural shows each year and it is quite feasible that animal contact may have occurred at one of these shows resulting in either concurrent infection of both farms with the same BVDV strain or one farm infecting the other at the show. As an adjunct to this, the authors are aware that Farm 40 has supplied colostrum to Farm 41 on several occasions; another potential route for transmission (this practice has now ceased consequent to our advice).

Whilst Farms 56 & 52 and 20 & 43, initially appear to have a common BVDV isolate upon 5′UTR phylogenetic and nucleotide analysis, this is less well supported by analysis of the N^pro^ region. No direct animal movements were identified to link these farms. Upon initial investigation, there was no common link between Farms 56 and 52 since they were more than 400 miles apart. However, both use embryo transfer services which could explain a common source of infection although this would seem unlikely given the analysis of the N^pro^ regions of BVDV isolates from both premises.

## Discussion

The predominant BVDV sub-genotype identified on the forty infected farms investigated in this study was BVDV 1a. This was isolated from the four main regions sampled in this study. BVDV 1b was isolated on two premises; one in the southwest and one in the southeast of England. Whilst these findings are largely in agreement with those of Vilcek et al. [10,19], the data in this paper also identifies a likely set of type 1d and 1e isolates supporting the conclusions of Strong et al. [20] that the genetic diversity of BVD virus in the UK has increased since 1999. There is potential that the emergence of BVDV sub-genotypes not previously reported in the UK could be a symptom of increased trade of cattle/cattle products.

No type 2 BVDV isolates were identified during the investigations in this study and this is re-assuring since the vaccines currently available in the UK only confer good cross protection against type 1 strains. Isolated occurrences of BVDV 2 have been reported in the UK [36-40] but these are normally linked to cattle movements from abroad and do not yet appear to have become endemic.

Current literature suggests that there is a continuing increase in the diversity of the BVDV 1 sub-genotype [3,11-15]. When this diversity is considered alongside the data presented by Strong et al. [20] and also in this manuscript, the emergence of isolates previously unreported in the UK leads one to question whether this has consequences for UK BVDV control programmes. The vaccines currently available in the UK which offer foetal protection against BVDV were first developed 13 and 17 years ago [41,42] and, at the time, were shown to provide good cross protection against the circulating type 1 strains. Further studies may be necessary to demonstrate that cross protection against BVDV 1 is still extensive considering the identification of increasing numbers of circulating BVDV 1 sub-genotypes. Of course, phylogenetic analysis may not provide an accurate indication of antigenic cross reactivity. Challenge studies, antigenic testing and/or investigation of the E2 and NS2-3 sequences (and predicted structures) may be required to ascertain whether the available vaccines are still cross protective against all BVDV 1 sub-genotypes. In addition to the increasing diversity of the BVDV 1 sub-genotype, the emergence of “atypical” pestivirus strains within the UK could become a particular concern. Originating from Brazil, the “HoBi” like viruses have been reported to cause severe disease and pathology in cattle in Northern Italy [43,44]. It is thought that “HoBi” like viruses were most likely introduced into Europe via live vaccines manufactured with contaminated bovine serum [43]. Thought should be given to the threat that atypical pestiviruses such as “HoBi” represent to the UK herd since, in a national herd with limited immunity to strains outside of BVDV type 1, the effects could be severe. To this end, continued surveillance of BVDV outbreaks should be undertaken, as well as screening of products containing imported bovine serum and considerable care with the licensing and use of live vaccines.

The phylogenetic analysis performed on isolates presented in this manuscript utilises the Neighbour Joining method [25] which has been most commonly used in the published literature for analysis and classification of BVDV. The bootstrap figures for the main branches in both Figures 2 and 3 appear to offer low statistical support (<70%) for the tree structure. Essentially, this is a consequence of the large number of isolates with high identity contained in the trees and the resultant difficulty in consistently defining the order within each branch. The correlation between the groupings illustrated in both phylogenetic trees and defined by nucleotide sequence analysis provides good support for the tree structures in Figures 2 and 3.

From the dataset investigated, it is evident from both Figure 2 and Table 2 that investigation of the 5′UTR alone would cause some confusion regarding the sub-genotypes present since it was unclear in some instances whether isolates were BVDV 1a, 1c or 1j from sole analysis of the 5′UTR. However, analysis of the N^pro^ region confirmed that where this confusion occurred, the isolates were actually BVDV 1a. Furthermore, one could argue that the BVDV 1i branch highlighted in Figure 2 ought to be included in the bracket demarcating BVDV 1a (along with the 1c and 1j reference strains). Again, N^pro^ analysis was able to provide a more convincing definition of the isolates on this branch confirming them as likely BVDV 1i isolates. Becher et al. [5] has previously noted the limitations of using the 5′UTR and the increased statistical support afforded to phylogenetic trees generated based upon the N^pro^ region. It is however worth re-iterating this point again since many of the most recent publications sub-genotyping BVDV isolates still concentrate on the 5′UTR with little additional information from other genomic regions. As a result, ideally, at least two regions of the BVDV genome should be analysed and agreement sought between them in order to define the isolates investigated with some certainty. Several studies have explored the use of three or more regions of the BVDV genome for sub-genotyping [45,46]. The methods employed by Liu et al. [46] produced a reliable definition of the isolates investigated and these might be employed to produce, unequivocally, a set of reference strains for future phylogenetic analyses. The analyses conducted in the current manuscript indicated that the confusion in sub-genotype classification could be further enhanced depending upon which reference strains were included. As a result, it would seem sensible to suggest that an agreed database of reference strains (agreed by the pestivirus community) is constructed to enable reliable and consistent analysis that is comparable between publications. A regulated reference strain library would also help avoid the confusion caused by the assignment of BVDV 1l to multiple unique BVDV isolates by Jackova et al. [11] and Kadir et al. [12]. Peterhans et al. [3] has subsequently suggested that the Turkish BVDV 1l strains be re-classified to BVDV 1p. However, Xue et al. [14] have already described a Chinese BVDV 1p sub-genotype. These matters require some clarification to ensure clarity with future sub-genotype definition.

In addition to the more traditional use of phylogenetics to investigate and type viral isolates present in a region or country, the data presented in this manuscript links viral isolates to the farms investigated and explores the potential routes of viral transmission. The farm level data collected enabled a detailed analysis of animal movements to be performed between premises which could, in some cases, be related directly to the phylogeny of the viruses isolated. In effect we are less concerned with primary branching and definition of viral type but instead interested in the clade formation at the end of each branch. Observing clustering at branch ends on the phylogenetic trees enables rapid identification of isolates with a high identity and thus high chance of a common infection route. Three different situations were described in the results:

Same farm, same isolate: Where more than one PI is isolated on the same farm, typing of those isolates commonly shows that the virus infecting each PI will share close to 100% nucleotide identity in both the 5′UTR and N^pro^ regions. The phenomenon of clustering of strains on the same farm is not new and has been noted previously [47]. As yet, the reason why such homology is observed within a herd, yet variation is seen between herds is unknown. Uninhibited viral replication in an infected foetus prior to the development of immunocompetance would seem an obvious place for genetic variation to occur, but it seems that this is not the case [48].

Same farm, different isolate: Where the one farm demonstrates two (or more) markedly different groupings upon phylogenetic analysis, it is likely that more than one infection event has occurred (perhaps two PIs bought in from different locations) producing clusters of PIs with two unique viral isolates.

Different farm, same isolate: Ridpath et al. [49] used sequence and phylogenetic analysis to confirm that the same strain of BVDV2 was responsible for multiple outbreaks between 1993 and 1995, however the routes of transmission were not investigated. For the first time, we show that phylogenetic and sequence analysis of BVDV isolates can be applied to determine factors influencing the epidemiology and transmission of the disease. Whilst somewhat limited, the network of farms involved in this research has enabled novel analysis of animal movement data alongside BVDV phylogeny. Where viral isolates from different farms were seen to cluster closely in both phylogenetic trees (with 99–100% nucleotide identity in both the 5'UTR and Npro regions), we identified links between the farms related to animal movements as demonstrated by Farms 33 and 34. There is a high frequency of animal movements within the network that includes these two premises and it is highly likely that either direct animal movements between these farms, or indirect movements through Farm 32 have resulted in the transmission of infection between Farms 33 and 34. There is perhaps an outstanding question regarding the status of Farm 32, especially if it is considered likely that infection has moved through this unit between Farms 33 and 34. At the point infection was detected on Farms 33 and 34, Farm 32 was being used as a facility to store heifers before they went on to the unit where they were destined to milk. If these animals were kept as separate management groups when on Farm 32, it is conceivable that the infection did not spread amongst the homebred milking herd and youngstock. This highlights the importance of taking representative samples from each management group when performing youngstock “spot tests” for BVD sero-surveillance [50]. Given the potential failure to diagnose infection on Farm 32, the other farms integrated into the movement network with Farms 33 and 34 were at an increased risk of infection before the PI animals were removed and biosecurity advice given. Had infection spread on Farm 32, a particularly high risk period would have been in 2008 when the unit was closed and animals transferred to other premises in the network. Animal movements from infected farms do not always result in infection of the destination premises but, where this does occur, we have shown that phylogenetic analysis may be used to determine the infection source. This may have implications in legal cases where typing of the viral isolates identified on the premises involved may prove or disprove that one farm infected another. Showing cattle has also been identified in this manuscript as a potential source of BVDV transmission. Expansion of an analysis of the sort described here with detailed animal movement and farm location data could allow for quantification and ranking of risk factors associated with BVDV transmission.

In conclusion, the work in this study supports the findings of Strong et al. [20] and has identified an additional isolate (BVDV 1d) that has not previously been reported in the UK. The apparently increasing diversity of strains in the UK may have consequences for vaccine efficacy and further work is required to determine whether this is the case. Whilst the strains identified in this research were all members of the BVDV 1 genotype, the increasing diversity highlights the need for continued and thorough surveillance in order to rapidly detect and react to incursions of new and atypical pestiviruses.

We have demonstrated that in depth investigations of animal movements and contacts between farms combined with phylogenetic analysis of BVDV can produce a “papertrail” of infection. This may have potential when utilised on a wider scale to quantify the risk factors associated with BVDV transmission between premises. These investigations may also have implications in legal cases where the origin of infection is under dispute.

Finally, the analysis of the data presented in this paper has demonstrated areas of pestivirus classification that require clarification. Particular issues arising from this work have been highlighted which would benefit from set guidelines in order to standardise future BVDV phylogenetic analyses. This is particularly important where these processes are used to determine the existence of new pestivirus strains/subgroups. This research, as a consequence, may also develop discussion within the pestivirus community in order to develop agreed guidelines to attain consistent investigation and classification for future epidemiological studies and newly identified isolates.

## Competing interests

The authors declare that they have no competing interests.

## Authors’ contributions

RB conducted the sample collection from herds 1–42, performed RT-PCR followed by sequence and phylogenetic analysis on samples from all farms involved. CT and LA gave extensive advice on the technical aspects of the phylogenetic and sequence analysis. CT, LA, GG and JB contributed to the drafting of the manuscript. All authors read and approved the final manuscript.
